# Epidemiological Analysis on the Occurrence of *Salmonella enterica* Subspecies *enterica* Serovar Dublin in the German Federal State Schleswig-Holstein Using Whole-Genome Sequencing

**DOI:** 10.3390/microorganisms11010122

**Published:** 2023-01-03

**Authors:** Silvia García-Soto, Jörg Linde, Ulrich Methner

**Affiliations:** Institute of Bacterial Infections and Zoonoses, Friedrich-Loeffler-Institute, Naumburger Str. 96a, 07749 Jena, Germany

**Keywords:** whole-genome sequencing, *Salmonella* Dublin, cattle, epidemiology

## Abstract

The cattle-adapted serovar *Salmonella* Dublin (*S*. Dublin) causes enteritis and systemic diseases in animals. In the German federal state Schleswig-Holstein, *S*. Dublin is the most important serovar in cattle indicating an endemic character of the infection. To gain information on dissemination and routes of infection, whole-genome sequencing (WGS) was used to explore the genetic traits of 78 *S.* Dublin strains collected over a period of six years. The phylogeny was analysed using core-genome single nucleotide polymorphisms (cgSNPs). Genomic clusters at 100, 15 and 1 cgSNPs were selected for molecular analysis. Important specific virulence determinants were detected in all strains but multidrug resistance in *S.* Dublin organisms was not found. Using 15 cgSNPs epidemiological links between herds were identified, clusters at 1 cgSNPs provided clear evidence on both persistence of *S.* Dublin at single farms in consecutive years and transmission of the organisms between herds in different distances. A possible risk factor for the repeated occurrence of *S.* Dublin in certain districts of Schleswig-Holstein might be the spreading of manure on pastures and grassland. Effective control of *S.* Dublin requires farm-specific analysis of the management supplemented by WGS of outbreak causing *S.* Dublin strains to clearly identify routes of infection.

## 1. Introduction

*Salmonella enterica* subspecies *enterica* serovar Dublin (*S.* Dublin), the cattle-adapted serovar of the genus *Salmonella* (*S.*) causes enteric and/or systemic diseases in bovines and, rarely also in other animal species and humans [1,2]. In Germany, ca. 120 outbreaks of salmonellosis in cattle are officially confirmed by a competent authority each year [3]. Outbreaks by the dominating serovar *S.* Typhimurium with a share of ca. 50% are recorded in nearly all federal states with cattle farming. *S.* Dublin also causes a high share of ca. 30–40% of all outbreaks, however, their occurrence is mainly concentrated on certain regions although single outbreaks are also detected in different federal states and in different years [3,4]. In the federal state Schleswig-Holstein, *S.* Dublin is by far the most important serovar in cattle and responsible for ca. 80–90% of the confirmed *Salmonella* outbreaks each year [3]. The repeated occurrence of *S.* Dublin infections in cattle farms over longer periods and in the same regions indicates an endemic character of the serovar in certain districts of the federal state. To enhance information on the routes of infection as a prerequisite for better control, genetic traits of the outbreak causing *S.* Dublin strains in different regions over a period of six years in the federal state Schleswig-Holstein should be analysed. Whole-genome sequencing (WGS) has been successfully applied for the characterisation of *S.* Dublin organisms from different origin [4,5,6,7,8,9]. Core-genome single nucleotide polymorphisms (cgSNPs)-based typing has shown a high discriminatory potential to identify closely related and non-related isolates [4,5,6,7,8,9,10]. WGS data also give information on virulence genes and antimicrobial resistance determinants of the isolates. The study aimed to gain information on the epidemiology of *S.* Dublin in cattle in a region with endemic occurrence of this serovar. WGS and bioinformatics analysis were used (i) to detect resistance and virulence markers within the *S.* Dublin genomes, (ii) to perform in-depth genotyping of the strains using hierarchical clustering based on cgSNPs and, (iii) to conclude on potential measures to improve future control strategies.

## 2. Materials and Methods

### 2.1. Bacterial Strains

The analysed data set comprised 78 *S.* Dublin strains from the collection of bovine-derived *Salmonella* organisms at the National Reference Laboratory (NRL) for Salmonellosis in cattle in Germany. Strains were obtained between 2016 and 2021 each from single confirmed outbreak episodes occurring in the German federal state of Schleswig-Holstein (SH) within the districts of Nordfriesland, Schleswig-Flensburg, Dithmarschen, Rendsburg-Eckernfoerde, Steinburg, and Pinneberg.

### 2.2. Phenotypic Characterisation: Serotyping and Antimicrobial Susceptibility Testing

*Salmonella* strains were phenotypically serotyped by slide agglutination test against anti-O and anti-H sera (SIFIN, Germany) according to the White-Kauffmann-Le Minor (WKL) scheme [11]. Broth microdilution using Sensititre EUVSEC plates (Trek Diagnostic Systems Ltd., East Grinstead, UK) was performed to determine the minimum inhibitory concentration (MIC). Epidemiological cut-off values given by the European Committee on Antimicrobial Susceptibility Testing (EUCAST) were applied to examine the antimicrobial susceptibility of the *S.* Dublin isolates to sulfamethoxazole (SMX), trimethoprim (TMP), ciprofloxacin (CIP), tetracycline (TET), meropenem (MERO), azithromycin (AZI), nalidixic acid (NAL), cefotaxime (FOT), chloramphenicol (CHL), tigecycline (TGC), ceftazidime (TAZ), colistin (COL), ampicillin (AMP), and gentamicin (GEN) [12].

### 2.3. DNA Preparation and Short-Read Sequencing

Genomic DNA extraction and purification were performed using Qiagen Genomic-tip 20/G kit (Qiagen, Germany). Previous to sequencing, Qubit Fluorometer and the double-stranded DNA (dsDNA) broad-range (BR) assay kit (Invitrogen, Waltham, MA, USA) was used to determine the concentration of the DNA. Sequencing libraries were prepared using Nextera XT DNA library preparation kit (Illumina, Inc., San Diego, CA, USA) and short reads paired-end sequencing of the *S.* Dublin strains was performed with MiSeq instrument (Illumina, Inc., San Diego, CA, USA) following the supplier’s instructions.

### 2.4. Initial Bioinformatics Analysis

The analysis of the sequenced data was carried on by the Linux-based bioinformatics pipeline WGSBAC v.2.2 (https://gitlab.com/FLI_Bioinfo/WGSBAC (accessed on 9 December 2022)). WGSBAC pipeline performs a complete genome data evaluation from the quality control of raw reads to the inference of bacterial phylogeny as published before [4,13]. Raw paired end reads from Illumina served as input for WGSBAC and its quality control was performed by FastQC v.0.11.7 [14]. Raw coverage was estimated as the number of reads multiplied by their average read length and divided by the genome size of the reference genome. Assembly of quality raw reads was performed by Shovill v. 1.0.4 [15] and the QUAST v. 5.0.2 [16] checked the quality of the assembled draft genomes. WGSBAC used the program Kraken 2 v. 1.1 [17] and the database kraken2DB to assign taxonomic labels to the sequenced reads and draft assemblies and to identify possible contamination. Raw coverage was estimated by a script adapted from https://github.com/raymondkiu/fastq-info (accessed on 9 December 2022). *Salmonella* serovar in silico prediction was performed by SISTR v.1.0.2 [18] and SeqSero2 [19] bioinformatics tools.

### 2.5. Screening of Antimicrobial Resistance (AMR) Genes, Plasmid Replicons, and Virulence Determinants

WGSBAC used ABRicate v. 0.8.10 [20] for the prediction of antimicrobial resistance (AMR) genes, plasmid replicons, and virulence determinants including *Salmonella* Pathogenicity Islands (SPIs). ABRicate works with the pre-downloaded databases ResFinder [21] and NCBI Bacterial Antimicrobial Resistance Reference (https://www.ncbi.nlm.nih.gov/pathogens/refgene/ (accessed on 9 December 2022)) for resistance genes detection, PlasmidFinder [22] for plasmid replicons screening, and Virulence Factor Database (VFDB) [23] for virulence factors prediction. Additionally, to identify further virulence factors, three customized databases for ABRicate were used: (1) “*SPIs*” for the screening of *Salmonella* Pathogenicity Islands (SPIs) (https://gitlab.com/FLI_Bioinfo_pub/spis_ibiz_database) (accessed on 9 December 2022) (2) “*pOU1115_dublin*” to search for the serovar-specific *S.* Dublin p0U1115 virulence plasmid sequence (GenBank accession no. DQ115388.2), and (3) “*spv_operon_dublin*” to identify the *S.* Dublin *spv* (*Salmonella* plasmid virulence) operon (*spv*RABCD). 60% coverage was utilized as the criterion to consider a strain positive for each gene sequence. Finally, the software AMRFinderPlus v. 3.6.10 [24] was used to detect chromosomal point mutations leading to AMR.

### 2.6. Strain Genotyping, Phylogeny, and Clustering

WGSBAC assessed the strains sequence type (ST) by classic Multilocus Sequence Typing (MLST) using the software mlst v. 2.16.1 [25] against the database PubMLST *Salmonella* MLST scheme (https://pubmlst.org/organisms/salmonella-spp (accessed on 9 December 2022)). For the identification of cgSNPs, WGSBAC used Snippy v. 4.3.6 [26] and mapped the query raw reads to the complete genome of *S.* Dublin strain 3246 (GenBank accession no. CM001151) used as reference. The generated cgSNPs alignment and cgSNP distant matrix were used for the phylogenetic reconstruction and clustering of the strains. The phylogenetic tree based on cgSNPs was annotated using RaxML v.8 [27]. The visualization of the phylogenetic tree was done using the interactive Tree of Life (iTOL) v. 4. Hierarchical clustering was computed using the R function hierClust v. 5.1 (https://search.r-project.org/CRAN/refmans/momr/html/hierClust.html (accessed on 9 December 2022)) based on the cgSNPs alignment and the cgSNP distance matrix created using the tool snps-dists v.0.63. According to the results of a previous study [4], a cut-off of 15 cgSNPs was applied to identify closely related strains. Furthermore, a broader cut-off of 100 cgSNPs was chosen to predict further clusters existing between the strains. In addition, clustering based on 1 cgSNPs was applied to detect clonal strains. To show the location of the strains, a geographical map was constructed using Ridom SeqSphere+. The strains were coloured by the hierarchical cluster under cgSNP 15 to which they corresponded.

## 3. Results

### 3.1. Phenotypic Characterisation: Serotyping and Antimicrobial Susceptibility Testing

Conventional serotyping revealed the antigenic formula of serovar Dublin (1, 9, 12: g, p; -) for all isolates. At phenotypic level, all 78 *S.* Dublin strains were susceptible to the antimicrobial substances tested. The MIC values (μg/mL) of the *S.* Dublin organisms were as follows: sulfamethoxazole (128–256), trimethoprim (<0.25–1), ciprofloxacin (0.03–0.06), tetracycline (4–8), meropenem (0.03–0.06), azithromycin (8–16), nalidixic acid (8–16), cefotaxime (<0.25), chloramphenicol (<8–8), tigecycline (1–2), ceftazidime (<0.05), colistin (1–2), ampicillin (<1–2) and gentamicin (1–2).

### 3.2. Genomic Characteristics of S. Dublin from Schleswig-Holstein

WGS of the 78 *S.* Dublin isolates from Schleswig-Holstein revealed an average of 1,690,056 reads per sample (range, 613,914 to 3,683,924 reads) and a read coverage of 80-fold on average (range, 33-to 169-fold). The assembled genomes consisted of an average of 34 contigs (range 25 to 224 contigs) and the genome size of the strains was 4,882,272 bp on average (range 4,853,805 to 5,153,704 bp) (Table S1). WGSBAC’s bioinformatics tools classified the genomes as *Salmonella enterica* species, the serovar Dublin was confirmed by in silico serotyping (Table S2).

### 3.3. Genomic Markers for Antimicrobial Resistance, Plasmids, and Virulence of S. Dublin from Schleswig-Holstein

Acquired resistance genes or point mutations were not detected among the 78 *S.* Dublin genomes from Schleswig-Holstein (Table S3). Only the chromosomal encoded gene *aac(6′)-Iaa* for aminoglycoside resistance as well as the multidrug-resistant efflux genes *mdsB* and *mdsA* were found in all strains (78/78). Genes encoding for response to stress through metal resistance, i.e., to gold, copper (*golT*, *golS*) or iron (*iroB*, *iroC*) were found as well in the 78 *S.* Dublin strains. Furthermore, all isolates contained the gene *fieF* encoding for the cation-efflux transporter and *sinH* for the intimin-like inverse autotransporter, both associated with virulence. Additionally, one strain (strain 3452) contained the gene *msr(C)* encoding for the ABC-F type ribosomal protection protein Msr(C) conferring resistance to macrolides. Plasmid replicons found among the 78 *S.* Dublin strains include IncFII(S) (78/78), IncX1 (78/78), IncFIB (AP001918) (2/78), ColRNAI (2/78) and IncFIC (FII) (1/78) (Table S4). Moreover, all the isolates were positive for screening of the *S.* Dublin p0U1115 plasmid sequence (GenBank accession no. DQ115388.2) encoding the *spv* virulence locus. Regarding genetic markers for virulence, most strains (68/78) contain a set of 109 virulence genes, followed by a minority of samples containing 108 (10/78). The missing genes include the *invJ* encoding for the type III secretion system needle length regulator InvJ (6/10), the *sspH2* encoding the type III secretion system effector SspH2 E3 ubiquitin ligase (3/10), and the *spvB* encoding the type III secretion system effector SpvB ADP-ribosylation activity (1/10). A total of eleven *Salmonella* pathogenicity islands (SPIs), SPIs 1, 2, 4, 5, 9, 13, 14, 16, 17, 19, and CS54 were found in most of the strains (49/78). Additionally, three strains (2309, 2521, 2534) contained SPI-12. A different SPI pattern was observed for 26/78 *S.* Dublin strains that lack at least one or a combination of more than one SPIs including SPI-1 (6/78), SPI-2 (1/78), SPI-4 (3/78), SPI-9 (1/78), SPI-13 (4/78), SPI-16 (6/78), SPI-17 (1/78), SPI-19 (4/78), CS54 (6/78) (Table S5).

### 3.4. Phylogeny of S. Dublin Strains in Schleswig-Holstein

**Figure 1:**
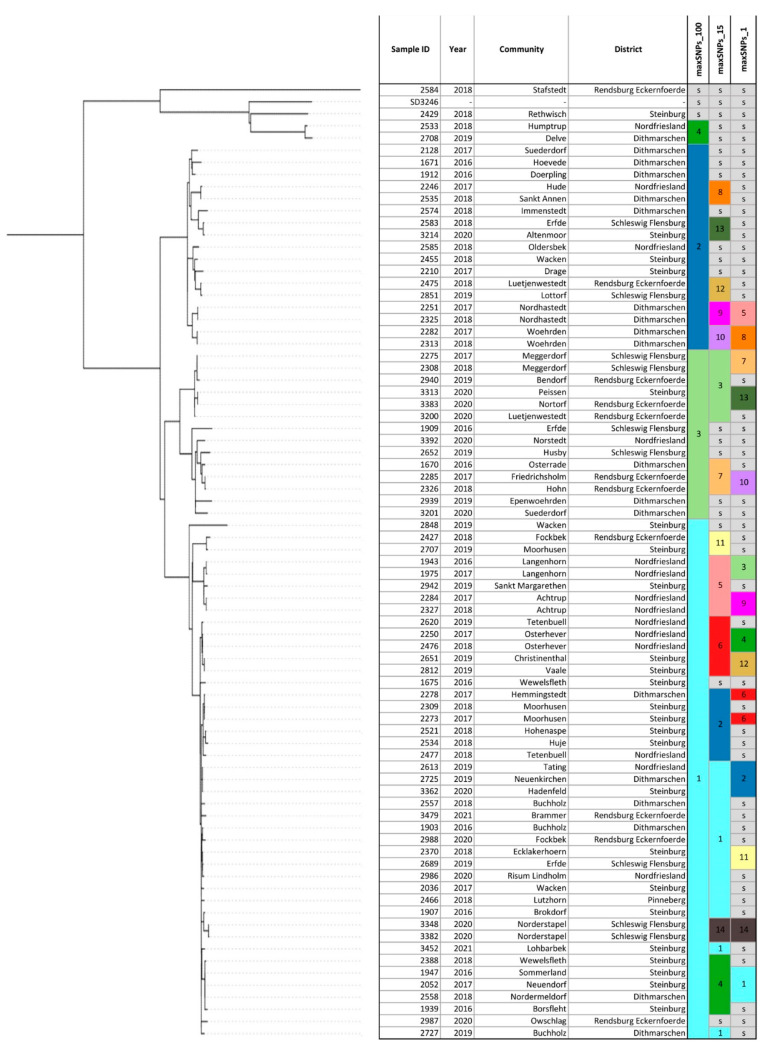
Phylogenetic tree based on core-genome Single Nucleotide Polymorphisms (cgSNPs) of the *S*. Dublin strains from Schleswig-Holstein. The legend shows the hierarchical clusters that group the strains under cut-offs of 100 cgSNPs, 15 cgSNPs, and 1 cgSNPs. Strains which are not clustered under a given cut-off are presented as singletons (s). The metadata attached (year and geographical location) adds information about the epidemiological context of the strains.

**Figure 2:**
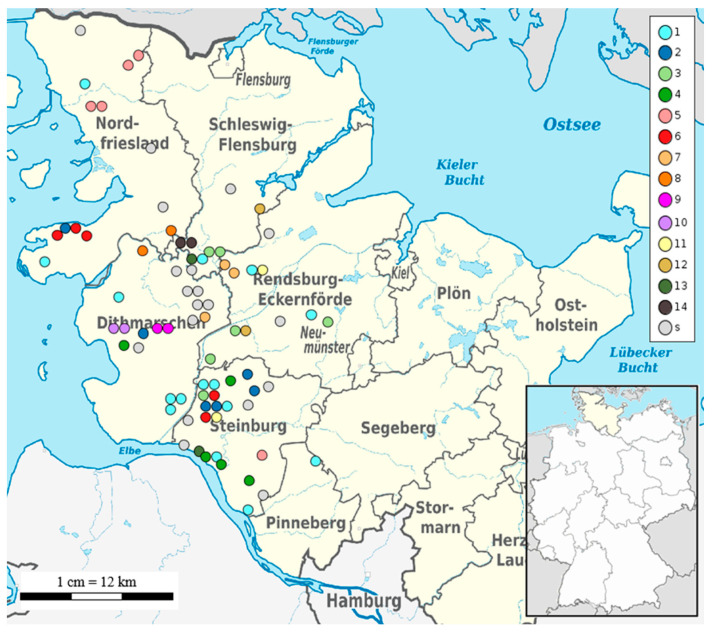
Geographical map of Schleswig-Holstein and its respective districts. Each coloured dot corresponds to an isolate placed according to the geographical coordinates of the sampling herd. The colours indicate the cluster numbers using 15 cgSNPs as cut-off. Strains which are not clustered are presented as singletons (s).

In silico multilocus-sequence typing (MLST) assigned sequence types (STs) for all *S.* Dublin isolates from Schleswig-Holstein. ST10 (77/78) was found in all strains except for strain 2584 isolated in 2018 in the district Rendsburg-Eckernfoerde which was typed as ST3734 (Table S2). For high-resolution genotyping, phylogeny based on cgSNPs was used. The size of the cgSNP alignment of the 78 strains from Schleswig-Holstein was 1559 bp. Pairwise cgSNP distance between the strains was on average 77 cgSNPs (range, 1 to 438 cgSNPs). The phylogenetic tree based on cgSNPs showed mainly closely related strains with the exception of five outliers: strains 2429, 2533, 2584, 2708, and the reference strain (*S.* Dublin strain 3246) (Figure 1). Clustering based on 100 cgSNPs separated all strains, except for two isolates (2429 and 2584) into four general clusters (Figure 1). Cluster 1 contained 43 strains from six different districts. The largest number of strains (18/43) originated from the district Steinburg followed by the district Nordfriesland (10/43). Cluster 2 contained 17 strains mainly from the district Dithmarschen (9/17), while three strains in cluster 2 were isolated in Steinburg or Nordfriesland. The majority of the total number of 14 strains in cluster 3 were detected in the districts Rendsburg-Eckernfoerde (5/14) and Schleswig-Flensburg (4/14), while strains from other districts were an exception. Clustering based on 15 cgSNPs revealed 14 different clusters containing 76% (59/78) of the strains, while 19 strains could not be clustered at this cut-off value (Figure 1). The largest cluster, cluster 1, contained 15 strains isolated between 2016 and 2021 in six different districts (Figure 2). This cluster contained two sets of clonal strains. The first clonal set comprised of three samples from three different districts: strain 2613 from Tating, strain 2725 from Neuenkirchen, and strain 3362 from Hadenfeld which were isolated between 2019 and 2020. Tating and Hadenfeld are about 70 km away from each other with Neuenkirchen roughly in between. Samples from the second clonal set were isolated in 2018 (strain 2370 from Ecklakerhoern, district Steinburg) and 2019 (strain 2689 from Erfde, district Schleswig-Flensburg) (Figure 1, Table S6).

No clonal relationship of the remaining eight strains in cluster 1 was detected, meaning that they are more than 1 cgSNP but less than 15 cgSNPs distant from each other. Additionally, all the remaining clusters described hereafter contained apart from clonal strains, non-clonal *S.* Dublin isolates in the range between 2–15 cgSNPs. Cluster 2 based on 15 cgSNPs contained six strains that were detected between 2017 and 2018 mainly from the district Steinburg (4/6) (Figure 2).

Two strains were found in other districts (strain 2278 in Dithmarschen, strain 2477 in Nordfriesland). Within cluster 2, two strains from one clone were isolated in 2017 in different districts: strain 2273 in Moorhusen (district Steinburg) and strain 2278 in Hemmingstedt (district Dithmarschen). These communities are 57 km away from each other. Cluster 3 also contained six strains (Figure 1) isolated between 2017–2019 mainly in the district Rendsburg-Eckernfoerde (3/6) and the neighbouring district Schleswig-Flensburg (2/6) (Figure 2). Two sets of clonal strains (Table S6) were part of cluster 3. The first clonal set comprised two strains (2275 and 2308) isolated in Meggerdorf in 2017 and the subsequent year. The second clonal set consisted of two strains from 2020: strain 3313 was detected in Peissen and strain 3383 in Nortorf. In fact, the sampling sites of both strains are 27 km away. Cluster 4 consisted of four strains from Steinburg and one strain from Dithmarschen isolated between 2016 and 2018. Strain 2558 from the district Dithmarschen and strains 1947 and 2052, both from Steinburg, were clonal (Figure 1, Table S6). While the sampling sites of strains 1947 and 2052 were only 14 km distant, strain 2558 was isolated about 75 km away from strain 1947. Four out of five strains grouped within cluster 5 originated from the district Nordfriesland, while strain 2942 belonged to district Steinburg (Figure 2). The strains 2284 and 2327 detected both in the community of Achtrup but in different years (2017 and 2018), were clonal (Table S6). Another clone (strains 1943 and 1975) was isolated in the community of Langenhorn also in successive years 2016 and 2017. Cluster 6 also contained five strains from the districts Nordfriesland and Steinburg only (Figure 2). One clone was detected in 2019 in the district Steinburg: Strain 2651 was found in Christinenthal and strain 2812 in Vaale which is 29 km distant (Table S6). Another clone (strains 2250 and 2476) was isolated in the community of Osterhever in the consecutive years 2017 and 2018. Three strains isolated between 2016 and 2018 formed cluster 7 based on 15 cgSNP. Strain 1670 was from district Dithmarschen. The other two strains isolated in the district Rendsburg-Eckernfoerde formed the same clone (Table S6). Strain 2285 was isolated in 2017 in a community only 7 km away from the place of origin of strain 2326 in 2018. The remaining seven clusters (8–14) identified using 15 cgSNPs, contained two strains each (Figure 2). Four of these clusters (8, 11, 12, 13, 14) contained pairs of non-clonal strains, while three clusters contained pairs of clonal strains. Clonal strains 2251 and 2325 in cluster 9 (Table S6) were isolated in the community of Nordhastedt (district Dithmarschen) in 2017 and 2018 (Figure 2). Additionally, cluster 10 consisted of two genomically identical strains isolated in consecutive years 2017 and 2018 in the community of Woehrden. Cluster 14 contained two strains (3348 and 3382) from one clone both isolated in 2020 in the community of Norderstapel (Schleswig-Flensburg).

## 4. Discussion

This study investigated 78 cattle-derived *S.* Dublin strains from confirmed outbreaks registered between 2016 and 2021 in the German federal state of Schleswig-Holstein (SH). Apart from three districts in the south of the federal state Bavaria [3], Schleswig Holstein is the region in Germany with an endemic occurrence of *S.* Dublin infections at cattle farms. A WGS-based characterisation of the *S.* Dublin organisms was performed to gain information on reasons for the repeated occurrence of the disease in SH. Although different *Salmonella* serovars cause infections or outbreaks in bovines and thus contribute to an increase in the antimicrobial resistance level in Europe in recent years [28], the host-adapted serovar *S.* Dublin does not seem to play a significant role. The analysis of the *S.* Dublin strains in this study showed a small number of genetic markers associated with antibiotic resistance. The antimicrobial susceptibility test as well as the analysis of the genomes did not reveal multidrug-resistant profiles within the *S.* Dublin isolates from Schleswig-Holstein. This supports findings of a previous analysis of *S.* Dublin strains originating from nearly all federal states in Germany [4] and also observations from other European countries [6,29]. In contrast, *S.* Dublin strains from both cattle and human beings coming from non-European countries such as the United States, Canada or China revealed a comprehensive antimicrobial resistance pattern [2,30,31,32,33]. The acquisition of plasmids that carry resistance genes such as IncA/C2, ColRNAI, or incompatibility (Inc) groups of plasmids have been identified in *S.* Dublin MDR isolates from cattle in the United States [34]. It is suggested that the difference between the European and American isolates is the carriage of the IncA/C2 plasmid [31]. Other genetic mechanisms identified include point mutations in resistance genes encoded in the chromosome (*gyr*A for fluoroquinolones and quinolones) and multidrug efflux pumps as the ones encoded by the *mdfA* gene [34]. Plasmid replicons associated with virulence such as IncFII(S) and IncX1 were the most predominant detected among the strains. In fact, all strains in this study carry the *Salmonella* virulence plasmid pOU115. The encoded *spv* operon is of special significance because of its role in the invasion of host cells [35]. Among other genetic markers for virulence, a quite stable and conserved pattern of 109 genes is carried by 68/78 strains followed by a minority of organisms (10/78) containing 108 genes. This underlines the pathogenic potential of *S.* Dublin strains circulating in the Schleswig-Holstein cattle population. Furthermore, most strains carry 10–12 SPIs and among them, SPI 1 is of special interest due to its role in the invasion of host cells. The detected lack of SPI-1 and the encoded gene *invJ* in six strains (2128, 1671, 1912, 2574, 2583, 3214) might affect their virulence [36] and, therefore, the clinical severity of the disease. Analysing the outbreaks concerned by studying the metadata of the farms might give both information on an epidemiological context between the farms and the virulence of the outbreak causing *S.* Dublin strains. Classical MLST (based on seven genes) typed all *S.* Dublin organisms in this study as ST 10, only one strain belonged to ST 3734. The only exceptional occurrence of others than ST 10 was also found in recent studies examining *S.* Dublin organisms from other German federal states [4,7] and is in line with the observation that ST 10 accounts for more than 90% of *S.* Dublin strains worldwide [32]. Therefore, regarding their antimicrobial resistance pattern, their pathogenic virulence potential but also their MLST profile the outbreak-causing *S.* Dublin strains from Schleswig-Holstein represent a homogeneous population which confirms findings from other European WGS-based *S.* Dublin analyses [4,5,6,7,8]. As the most strains of *S.* Dublin belong to ST 10, MLST is not an appropriate tool to further distinguish the strains. Therefore, in this study cgSNP typing and hierarchical clustering served as discriminatory tools to analyse the *S.* Dublin epidemiology in the cattle population of Schleswig-Holstein. SNP-typing based on whole-genome-sequencing has been proven to be a powerful tool to study *S.* Dublin phylogeny in Europe [4,5,6,7,8,9,10]. While a threshold of 15 SNPs was shown to cluster strains from the same herds [6], 40 and 100 SNPs were used to detect intermediate and general clusters [4]. In this study, clustering was applied to strains from a rather small area with an endemic occurrence of genomically homogeneous *S.* Dublin strains. With the aim to detect possible routes of infection and to gain information on the spreading of outbreak-causing organisms, clonally related strains were also identified. Although clonal strains are genomically identical (i.e., distance of 0 SNPs), in this study one cgSNP was used as cut-off for clustering to take into account potential sequencing errors. In fact, general clustering using 100 SNPs as threshold grouped the *S*. Dublin organisms to different regions with predominant occurrence over longer periods, indicating that the *S*. Dublin strains are not distributed evenly over the entire region in question. Whereas one cluster was detected in nearly all regions of the federal state, two further clusters were located in only two districts each, suggesting a different pattern of transmission of the strains. As clustering of *S.* Dublin isolates using a threshold of 15 cgSNPs indicates an epidemiological link between herds [4,37], this cut-off value was also used in this study. Numerous clusters proved epidemiological links between cattle farms in a rather close distance but also in faraway regions in short and long periods. These findings clearly indicate both persistence and transmission of outbreak causing *S*. Dublin strains at or between different farms in a relatively close distance but also at farms located in districts far from each other. Despite these indications on the dissemination of closely related strains, clear evidence on the persistence of *S.* Dublin at a single farm or the transmission of the organism between herds in different distances can only be given by identifying clonal strains using a threshold of 1 cgSNPs. The repeated occurrence of identical clones in the same herds in consecutive years gives evidence for a long-term infection or persistence of the strain, respectively. Persistence of *S.* Dublin might occur (i) at the farm after insufficient cleaning and disinfection, (ii) in the direct environment of the farm, (iii) at pastures after grazing by infected animals or spreading of contaminated manure or (iv) by unidentified persistently infected carrier animals. Reasons for persistence at farms are complex and can only be analysed by comprehensive sampling followed by ascertaining the operations at the farm. The transmission of *S.* Dublin between farms is also due to different factors. A transfer of identical *S.* Dublin strains between nearby farms might be caused, e.g., by close contact between animals, joint use of equipment, shared use of pastures, etc. The most important reason for the transmission of clonal *S.* Dublin organisms between distant and far distant farms is with a high probability the trade of infected animals [4]. Compared with ubiquitous occurring *Salmonella* serovars, the cattle-adapted serovar *S.* Dublin is highly animal associated and only occasionally detected in a non-bovine environment. Therefore, findings of *S.* Dublin in previously not affected farms should first raise questions whether the entry might be due to infected animals from other cattle farms. Furthermore, a possibly underestimated risk factor for the repeated occurrence of *S.* Dublin outbreaks at cattle farms in certain districts of Schleswig-Holstein might be the manure management. Spreading of manure on pastures and grassland after the first grass harvesting in spring followed some weeks later by grazing of juvenile animals, pregnant heifers and cows or by further harvesting of grass as well as production of silage might result in maintaining the *S.* Dublin infection in the herds. It is also not excluded that manure of single farms is not only spread on farm-associated pastures but also on pastures of other farms which might be located in the same but also in far distant districts. Therefore, in regions with repeated occurrence of *S.* Dublin outbreaks also the manure management should be considered in the analysis of the infection. Apart from effective biosecurity procedures to control *Salmonella* infections at cattle farms [1,3,7], WGS is an valuable tool to analyse outbreak-causing *S.* Dublin organisms and also strains from other serovars [6,8,10,30,32]. The identification of both, strains with a link between farms and identical strains in different herds using various thresholds for cgSNPs analysis is highly important to detect routes of infection and represents the basis for effective intervention measures. Conclusions to combat *S.* Dublin infections in an endemic region require not only information on the causing strains involved but also metadata on the farms in question. The epidemiological analysis of both parameters is crucial to interrupt both persistence and transmission of *S.* Dublin at and between single farms in close regions but also in larger areas with endemic occurrence of the organisms. Not only the well-known ban of cattle movement from infected to non-infected herds [4,6,37] but also measures for modifying the management at farms, improvement of biosecurity, management of manure, the manner to use pastures, production and use of feed at different farms, and methods to identify persistent carriers need to be established. Therefore, future strategies to control or eradicate *S.* Dublin especially in endemic areas should consider WGS of all outbreak-causing strains to complement the analysis of the existing infection routes at and between farms.

## Data Availability

The raw sequencing data from our previous study (10 *S.* Dublin strains) [4] can be assessed under the BioProject accession number PRJNA678856 (https://www.ncbi.nlm.nih.gov/bioproject/?term=PRJNA678856 (accessed on 9 December 2022)). The new raw sequencing data generated for this study (68 *S.* Dublin strains) was deposited in ENA under the Project PRJEB56661.

## Supplementary Materials

The following supporting information can be downloaded at: https://www.mdpi.com/article/10.3390/microorganisms11010122/s1, Table S1: General genomic characteristics of *Salmonella* Dublin from Schleswig-Holstein; Table S2: Genotyping and serotyping results of *Salmonella* Dublin from Schleswig-Holstein; Table S3: AMR genes screening of *Salmonella* Dublin from Schleswig-Holstein; Table S4: Plasmid replicons screening results of *Salmonella* Dublin from Schleswig-Holstein; Table S5: Virulence genes, SPIs, virulome screening results of *Salmonella* Dublin from Schleswig-Holstein; Table S6: SNPs matrix distance between genomes of *Salmonella* Dublin from Schleswig-Holstein.
